# Dual trends in physical fitness and suspected myopia before and during the COVID-19 pandemic: a single-school study of children aged 6–11 years in rural Zhejiang, China

**DOI:** 10.3389/fpubh.2026.1810566

**Published:** 2026-07-08

**Authors:** Haolu Shu, Qianhui Zhou, Taiji Matsui, Norikazu Hirose, Akira Maehashi, Jianhai Lei, Linjing Jiang

**Affiliations:** 1 Faculty of Sport Sciences, Waseda University, Saitama, Tokorozawa, Japan; 2 Graduate School of Sport Sciences, Waseda University, Tokorozawa, Saitama, Japan; 3 Physical Education Department, Wen Yuan of Lishui City School, Lishui, Zhejiang, China; 4 Institutes of Innovation for Future Society, Nagoya University, Nagoya, Japan

**Keywords:** children, Chinese, COVID-19, myopia, physical fitness

## Abstract

**Introduction:**

Social distancing and reduced physical activity during the COVID-19 pandemic may have adversely affected children's physical fitness and visual health. This study compared physical fitness indicators and the prevalence of suspected myopia among Chinese children aged 6–11 years in 2019 (pre-pandemic) and 2020 (during the pandemic) using cross-sectional school screening data.

**Methods:**

Data were collected in 2019 (*N* = 1,183) and 2020 (*N* = 1,120), and analyzes complete cases for each outcome (maximum *n* = 1,069 and *n* = 1,116, respectively). Physical fitness outcomes included body mass index (BMI), flexibility, and athletic performance. Visual acuity (unaided distance) was used to define suspected myopia as visual acuity < 5.0 (Chinese standard logarithmic chart) in either eye. Year differences were evaluated using regression models adjusted for age and sex with class-level cluster-robust standard errors. Prevalence ratios for suspected myopia were estimated using modified Poisson regression, and multiple tests were controlled using the Benjamini–Hochberg false discovery rate.

**Results:**

During the pandemic period, BMI increased among children aged 6–8 years. Flexibility declined among 6-year-olds and girls aged 7 years. Rope-jumping performance improved in most age groups, except for boys aged 6 and 10 years, while no statistically significant changes were observed in 50 m sprint performance across age groups. The prevalence of suspected myopia increased significantly in 2020 compared with 2019, with age- and sex-specific differences, including higher risks among girls aged 7 and 11 years and boys aged 10 years.

**Discussion:**

These findings highlight the need for targeted interventions—particularly for younger children—to mitigate the potential impacts of the pandemic on weight management, physical fitness, and visual health, considering age- and sex-specific differences.

## 1 Introduction

The World Health Organization declared COVID-19 a Public Health Emergency of International Concern on 30 January 2020 and characterized it as a pandemic on 11 March 2020 (1). Due to rapid spread of the disease, China's provinces, municipalities, and autonomous regions activated a Level 1 response to major public health emergencies (2). Lockdown or strict control measures were implemented across the country. Notably, the Ministry of Education announced that the semester from February 19 to June 16, 2020, would be conducted through home-based online classes (3, 4). The age range of 6–11 years represents a critical developmental window, during which children undergo rapid physiological growth and lifestyle habit formation (5). Behaviorally, this period is essential for establishing foundational movement skills and lifelong physical activity habits (6). Biologically, the neuromuscular system, cardiorespiratory capacity, and skeletal growth are highly plastic and responsive to environmental stimuli (7). Furthermore, from an ophthalmic perspective, 6–11 years is a sensitive phase for emmetropization and ocular development, where the eye structure is exceptionally vulnerable to visual stressors (8). However, the COVID-19 pandemic severely disrupted these developmental trajectories through interlinked behavioral and biological pathways.

As a result of home confinement, students reduced the time spent on physical activities, while increasing their screen time (9, 10). Behaviorally, the restriction of physical space and the shift to prolonged online learning dramatically increased sedentary behavior and displaced moderate-to-vigorous physical activity (MVPA) (11). Biologically, insufficient mechanical loading and cardiorespiratory stimulation may impair muscular and motor development, and reduced physical fitness during childhood may track into adolescence and adulthood (12, 13). Concurrently, the behavioral reduction in outdoor time combined with extended near-work screen exposure triggers biological mechanisms in ocular growth. Specifically, diminished exposure to natural sunlight reduces retinal dopamine release—a key inhibitor of axial elongation—thereby accelerating scleral remodeling and driving myopia progression (14, 15). Despite the importance of mastering physical fitness and protecting eyesight during this specific childhood stage, the COVID-19 restrictions have delayed the achievement of minimum proficiency in physical fitness, while possibly accelerating the progression of myopia.

Recent findings underscore that the COVID-19 pandemic significantly disrupted children's physical activity patterns and increased risk factors for health issues, such as reduced fitness and accelerated myopia progression (16). Not only can physical inactivity during childhood ultimately lead to diminished physical proficiency, but spending less time outdoors is also directly associated with a higher incidence of myopia. This is a key issue, resulting in reduced physical proficiency in adolescence, which can continue into adulthood (17, 18). Despite the importance of mastering physical fitness and protecting eyesight during childhood, the COVID-19 restrictions have delayed the achievement of minimum proficiency in physical fitness, while possibly accelerating the progression of myopia. Two studies have demonstrated the pandemic's negative effect on the motor development of schoolchildren aged 6–9 years (19, 20). Conversely, one study showed a positive effect of the pandemic on children's motor-skill development (21). Regarding myopia, one study demonstrated that home-based online learning accelerated the progression of myopia in elementary school students during the COVID-19 pandemic (22).

Despite the urgency of these issues, few studies have simultaneously examined both physical fitness and visual acuity trends in school-aged children across pre- and intra-pandemic periods. To address this gap, this study investigated changes in physical fitness indicators and myopia prevalence among Chinese children aged 6–11 years using repeated cross-sectional data from 2019 and 2020. By identifying age- and sex-specific patterns, the findings aim to inform future health and education policies, especially in preparedness for prolonged behavioral disruptions such as pandemics.

## 2 Methods

### 2.1 Study design and participants

This study was conducted in Lishui City (population ~2.5 million; area 17,298 km^2^), a rural municipality in Zhejiang Province, China. Data were obtained from two waves of school-based health and fitness screening conducted in 2019 (pre-pandemic) and 2020 (during the pandemic) among primary school students aged 6–11 years. This study used a fully anonymized secondary-use dataset derived from school-based health and fitness screenings conducted in 2019 and 2020 in Lishui City, Zhejiang, China. Because the data-providing school did not have a formal institutional ethics committee, the secondary analysis protocol was reviewed and authorized by the leadership of the school. This study involved a secondary analysis of fully anonymized school-based health and fitness screening data from children. The study protocol was also reviewed by the Waseda University Institutional Review Board for institutional compliance (SU29-22; Approval No. 2023-HN051). Clinical trial number: not applicable.

A local public primary school in a rural setting was selected as the study site based on feasibility during the pandemic and an existing collaboration with the research team. All enrolled students at the school who participated in the annual screening were included. Because broader-area random sampling was not feasible under pandemic-related restrictions, the study used a single-site, repeated cross-sectional design based on an existing anonymized database for 2019 and 2020. All raw records were anonymized by the data holder before release to the research team.

### 2.2 Measures

The National Student Physical Fitness Standard for primary school students in China was used to assess physical fitness, including 50 m sprint time(s), 1-min rope-jumping performance (number of jumps), and sit-and-reach distance (cm) (23, 24). Measurements were conducted by trained examiners (school staff and research assistants) following the standardized national protocol. To assess sprint time, a 100 m straight racetrack was used, along with a starting flag, whistle, and stopwatch. Participants began in a stationary standing position, with one foot in front of the other, and the front foot placed behind the starting line. Once the participant was motionless and ready, the starter issued the command “Set,” then blew the whistle and waved the starting flag to signal the timekeeper to start timing. Participants ran as fast as possible across the finish line, where the timekeeper stopped the stopwatch. Each participant was allowed two trials, and the best time was recorded to the nearest two decimal places.

Flexibility was assessed using the sit-and-reach test, which was performed using a seated forward flexion tester (GMCS-IV; Jianmin, Beijing, China). During the test, participants sat on a flat surface with their legs extended straight and positioned flat against the longitudinal plate of the tester, approximately 10–15 cm apart. While keeping their legs straight, participants bent their upper bodies forward to reach as far as possible. Each participant performed the test twice and the best score was recorded in centimeters (cm) to the nearest decimal place. To assess the children's coordination and muscle endurance, a 1-min rope-jumping test was conducted. Participants were instructed to skip continuously for 1 min using appropriately sized ropes, ensuring they landed on both feet with each jump. The number of skips was counted and recorded by the tester.

Anthropometric measurements included objectively measured height (cm) and weight (kg), recorded without shoes and in light clothing to the nearest 0.1 cm and 0.1 kg, respectively. BMI was calculated as weight (kg) divided by height squared (m^2^). Additional, age- and sex-standardized BMI-for-age *z*-scores (BAZ) were calculated according to the WHO 2007 Growth Reference for children and adolescents aged 5–19 years using the WHO reference standards (25).

Suspected myopia was defined based on unaided distance visual acuity measured with the Chinese standard logarithmic visual acuity chart, as VA < 5.0 in either eye (26). The same examiners, equipment, and measurement protocols were used in both 2019 and 2020.

### 2.3 Statistical analysis

Analyses were performed on the analytic sample after excluding records with missing values in the variables required for each outcome. Descriptive statistics were summarized by year (2019 vs. 2020), age (6–11 years), and sex, using mean ± SD for continuous variables and n (%) for categorical variables. For continuous outcomes (height, weight, BMI, 50 m sprint time, 1-min rope-jumping counts, and sit-and-reach distance), between-year differences were evaluated using ordinary least squares (OLS) regression. Year, age, and sex were treated as categorical factors, and a full factorial specification (Year × Age × Sex) was used to allow heterogeneous effects across subgroups. To account for within-class correlation in the school-based data, inference was based on class-level cluster-robust standard errors. For suspected myopia (binary outcome), prevalence ratios (PRs) comparing 2020 with 2019 were estimated using modified Poisson regression with a log link, using the same factorial specification (Year × Age × Sex) and class-level cluster-robust standard errors. Model-based marginal contrasts were used to obtain (1) 2020 vs. 2019 contrasts within each Age × Sex stratum, and (2) sex contrasts within each Year × Age stratum; two-sided *p*-values and 95% confidence intervals were reported. Multiple testing was controlled using the Benjamini–Hochberg false discovery rate (BH-FDR) procedure: (1) across primary outcomes for the overall year effect in the main models, and (2) within each outcome across the set of prespecified subgroup contrasts. Statistical significance was evaluated using two-sided tests with α = 0.05, with FDR-adjusted q-values reported where applicable. All analyses were conducted in Python (pandas, statsmodels). Sensitivity analyses were conducted for suspected myopia to assess the influence of age and sex compositional imbalance between waves. We repeated the analysis after excluding children aged 11 years and fitted weighted modified Poisson models (1) using stabilized inverse probability weights based on age, sex, and age × sex (27). We additionally applied post stratification weights to standardize both waves to the 2019 age and sex distribution (27–29). Weighted models used a log link and class level robust variance, consistent with the primary suspected myopia analysis.

## 3 Results

### 3.1 Students characteristics

**Figure 1:**
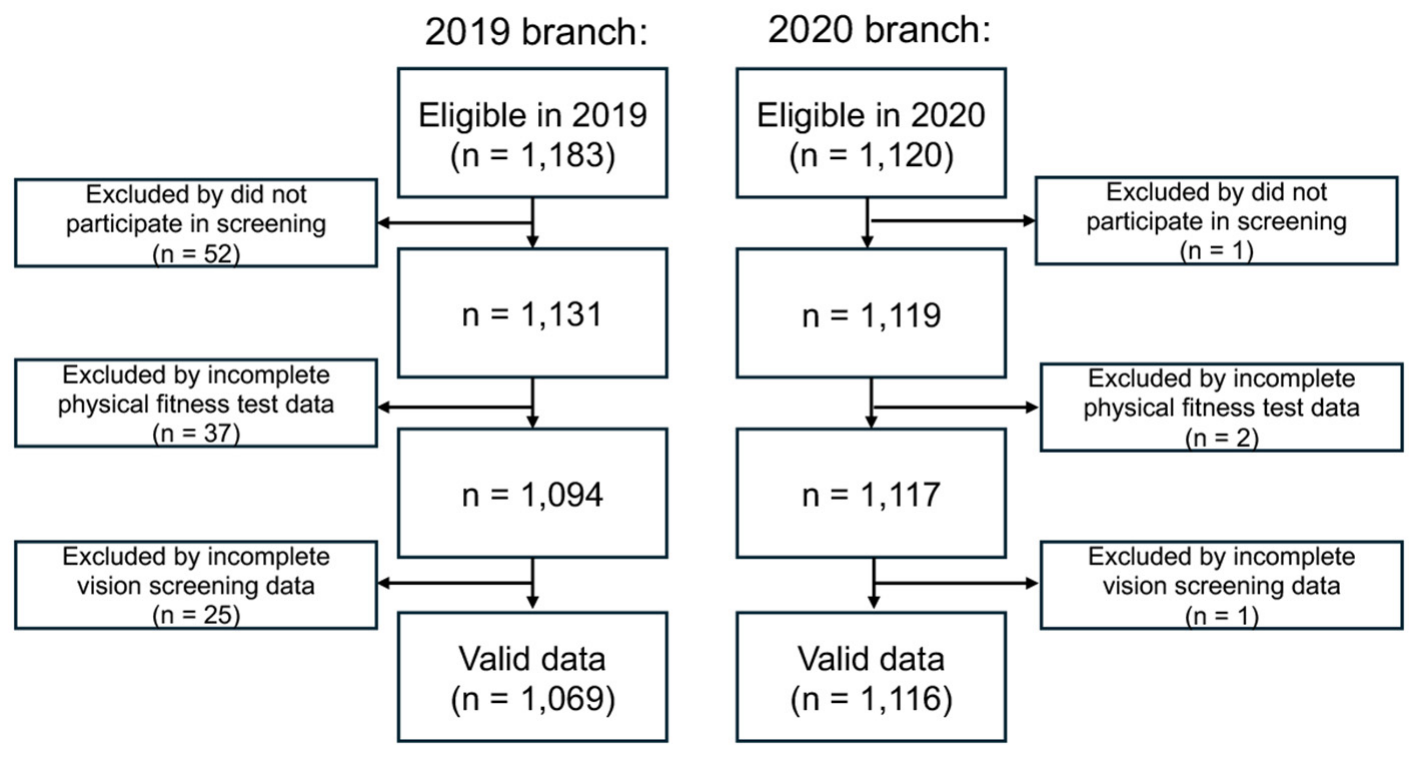
Flowchart of participant inclusion and exclusion.

A total of 1,183 and 1,120 children were screened in annually in November in both 2019 and 2020, respectively. Analytic sample sizes varied by outcome due to missingness, and the maximum complete-case sample was 1,069 (2019) and 1,116 (2020). For children aged 6–11, the sex distribution remained non-significant before and during the pandemic, except for 11-year-olds (*p* ≤ 0.037). Table 1 presents participant demographics. A participant flowchart illustrating inclusion, exclusion, and missing data handling is presented in Figure 1.

**Table 1:** Analytic participant characteristics.

Characteristic	Before (2019)		During (2020)		*P* value
	*n*	%	*n*	%	
Overall	1,069		1,116		0.880
Boys	602	56.3	634	56.8	
Girls	467	43.7	482	43.2	
Six years old	156		151		
Boys	87	55.8	81	53.6	0.708
Girls	69	44.2	70	46.4	
Seven years old	227		183		
Boys	113	49.8	103	56.3	0.190
Girls	114	50.2	80	43.7	
Eight years old	181		236		
Boys	102	56.4	123	52.1	0.390
Girls	79	43.6	113	47.9	
Nine years old	134		187		
Boys	77	57.5	102	54.5	0.604
Girls	57	42.5	85	45.5	
Ten years old	199		141		
Boys	128	64.3	82	58.2	0.249
Girls	71	35.7	59	41.8	
Eleven years old	172		218		
Boys	95	55.2	143	65.6	0.037
Girls	77	44.8	75	34.4	

### 3.2 Anthropometric parameters

**Figure 2:**
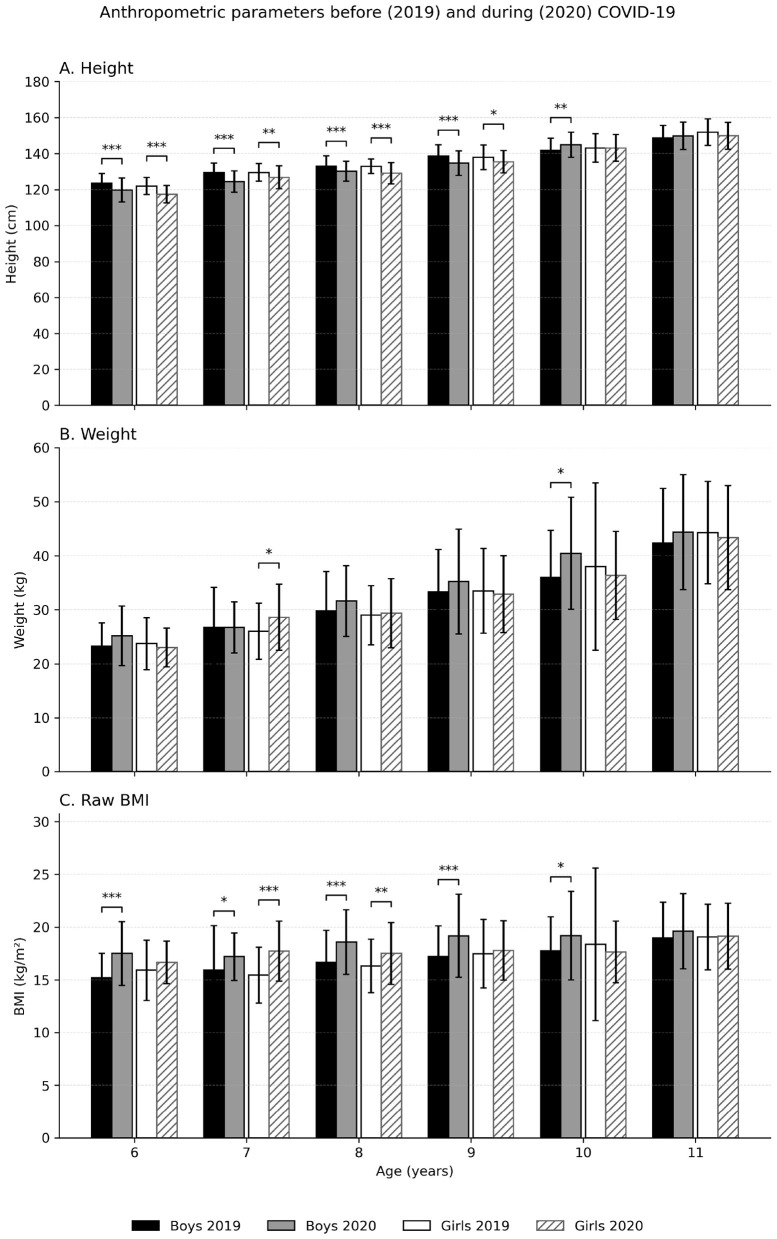
Age-specific changes in **(A)** height (cm), **(B)** weight (kg), and **(C)** body mass index (BMI; kg/m^2^) among boys and girls before (2019) and during (2020) the COVID-19 pandemic. Bars represent mean ± SD for each age group (6–11 years). Within each age group, bars are arranged from left to right as follows: boys 2019 (solid black), boys 2020 (solid gray), girls 2019 (white with black outline), and girls 2020 (hatched). Brackets with asterisks indicate significant differences between 2019 and 2020 within the same sex and age group (*q < 0.05, **q < 0.01, ***q < 0.001).

Model-based comparisons indicated clear between-year differences in anthropometric outcomes, with evidence that the magnitude of changes varied according to age and, for some outcomes, by sex. In the primary factorial models, year effects were statistically significant for height, weight, and BMI (all *p* < 0.001), with additional evidence of age- and/or sex-specific heterogeneity for height and BMI. Compared with 2019, mean height in 2020 was significantly lower across most younger age groups, including boys aged 6–9 years and girls aged 6–9 years (all FDR-adjusted *q* < 0.05). The largest reductions were observed among boys aged 7 years (−5.05 cm) and girls aged 6 years (−4.53 cm). In contrast, boys aged 10 years showed a significantly greater mean height in 2020 than in 2019 (+2.98 cm; *q* < 0.01; Figure 2A).

Between-year differences in weight were more limited. Significant increases were observed in girls aged 7 years (+2.61 kg; q < 0.05) and boys aged 10 years (+4.44 kg; q < 0.05), whereas no other age–sex strata showed FDR-significant differences (Figure 2B). BMI was consistently higher in 2020, particularly among younger children. Significant increases in BMI were observed in boys aged 6–10 years (+1.27 to +2.30 kg/m^2^; all *q* < 0.05) and girls aged 7–8 years (+1.17 to +2.25 kg/m^2^; all *q* < 0.05; Figure 2C).

**Figure 3:**
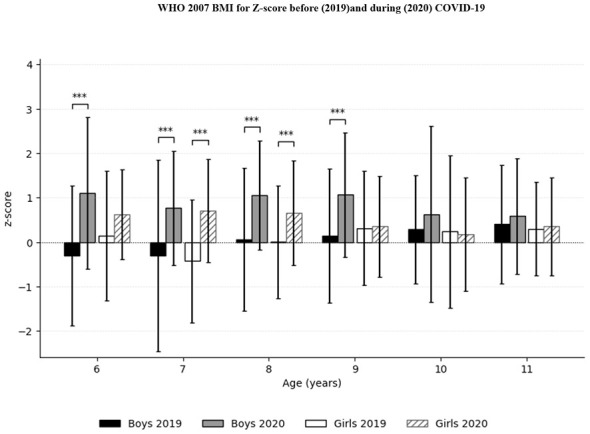
Bars indicate mean BMI-for-age z-scores and error bars represent standard deviations (SD). Asterisks denote significant differences between 2019 and 2020 within each age group (***P < 0.001).

WHO 2007 BMI-for-age *z*-scores also showed higher values in 2020, particularly among younger children. Significant increases were observed in boys aged 6–9 years (+0.83 to +1.51 SD units; all FDR-adjusted *q* < 0.001) and girls aged 6–8 years (+0.51 to +1.18 SD units; all *q* < 0.05). The largest increases were found in boys aged 6 years (+1.51 SD units) and girls aged 7 years (+1.18 SD units). No significant between-year differences in BMI *z*-scores were observed among older children aged 9–11 years in girls or 10–11 years in boys (Figure 3).

### 3.3 Physical fitness levels

**Figure 4:**
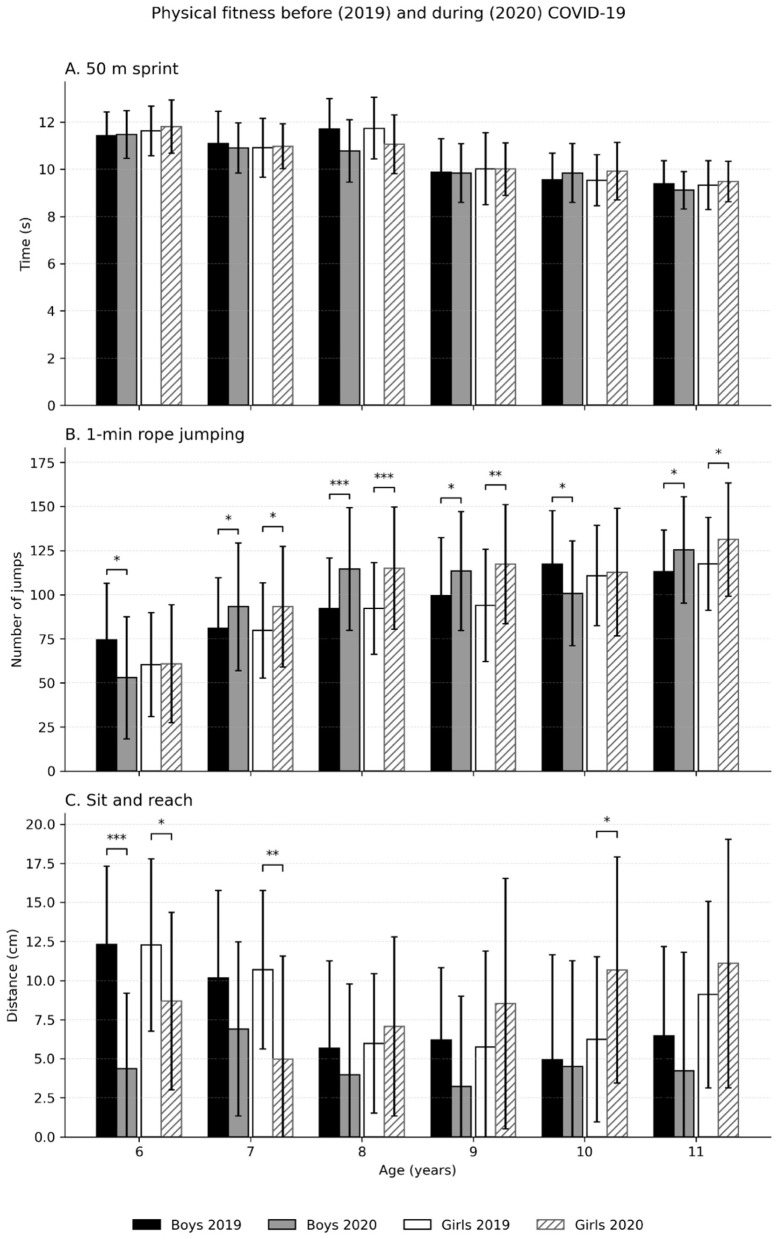
Age-specific changes in **(A)** 50 m sprint time (s), **(B)** 1-min rope-jumping performance (number of jumps), and **(C)** sit-and-reach distance (cm) among boys and girls before (2019) and during (2020) the COVID-19 pandemic. Bars represent mean ± SD for each age group (6–11 years). Within each age group, bars are arranged from left to right as follows: boys 2019 (solid black), boys 2020 (solid gray), girls 2019 (white with black outline), and girls 2020 (hatched). Brackets with asterisks indicate significant differences between 2019 and 2020 within the same sex and age group (*q < 0.05, **q < 0.01, ***q < 0.001).

The fitness outcomes showed domain-specific changes. In the primary factorial models, year effects were not statistically significant for the 50 m sprint time (*p* = 0.22), whereas significant year effects and age-dependent patterns were observed for the 1-min rope jumping and sit-and-reach tests (both *p* < 0.001), with evidence of effect heterogeneity across age, and across sex for flexibility and rope jumping. After multiplicity control within the outcome, no age–sex stratum showed a statistically reliable 2019–2020 difference in sprint time. A sex difference was observed at age 6 in 2020, with girls being slower than boys (+0.33 s; *q* < 0.01; Figure 4A). Clear year differences were observed, with improvements in 2020 for most ages but notable exceptions in the number of 1-min rope-jumps. Rope-jumping counts were higher in 2020 for ages 7–9 years in both sexes (approximately +12 to +23 jumps/min across strata; all *q* < 0.05), and higher at age 11 for both girls (+13.88) and boys (+12.19; both *q* < 0.05). In contrast, boys aged 6 and 10 years showed lower performance in 2020 (age 6: −21.41; age 10: −16.57; both *q* < 0.05; Figure 4B). Flexibility declined in 2020 among younger children (boys age 6: −7.94; girls ages 6–7: −3.59 and −5.71; all *q* < 0.05), whereas girls aged 10 years showed higher flexibility in 2020 (+4.44; *q* < 0.05; Figure 4C).

### 3.4 Suspected myopia

Overall, the prevalence of suspected myopia was higher in 2020 than in 2019 in the primary model (year effect *p* < 0.001), and the between-year contrast varied by age (year × age *p* < 0.01). After controlling for multiple comparisons within this outcome using the Benjamini–Hochberg false discovery rate (*q*-values), PRs comparing 2020 with 2019 were significantly higher among girls aged 7 years [PR = 1.83; 95% CI (1.29, 2.59); *q* = 0.009] and 11 years [PR = 1.41; 95% CI (1.10, 1.81); *q* = 0.020], and among boys aged 10 years [PR = 1.24; 95% CI (1.07, 1.43); *q* = 0.020]. In contrast, boys aged 6 years showed a lower prevalence in 2020 than in 2019 [PR = 0.65; 95% CI (0.49, 0.88); *q* = 0.020]. Several additional strata showed nominal increases (*p* < 0.05) but did not remain significant after FDR adjustment (Table 2). Sensitivity analyses addressing the sex composition imbalance at age 11 yielded similar conclusions. After excluding children aged 11 years, the overall age and sex adjusted prevalence ratio for suspected myopia comparing 2020 with 2019 remained above 1.0. Weighted analyses also produced estimates close to the primary age and sex adjusted model. Specifically, the stabilized inverse probability weighted model based on age × sex gave a PR of approximately 1.14, and post stratification to the 2019 age and sex distribution gave a PR of approximately 1.15. Detailed sensitivity results are provided in Supplementary Table S1.

**Table 2:** Age- and sex-specific prevalence of suspected myopia in 2019 (pre-pandemic) and 2020 (during the pandemic), and prevalence ratios (PRs) comparing 2020 vs. 2019.

Age	Sex	Pre 2019 (*N*/%)	During 2020 (*N*/%)	PR (2020 vs. 2019)	95% CI	*q*-value
6	Boys	87/47.13%	81/30.86**%**	0.65	[0.49, 0.88]	**0.020**
	Girls	69/44.93%	70/40.00**%**	0.89	[0.57, 1.39]	0.734
7	Boys	113/41.59%	103/40.78**%**	0.98	[0.76, 1.26]	0.877
	Girls	114/28.07%	80/51.25**%**	1.83	[1.28, 2.59]	**0.009**
8	Boys	102/51.96%	123/55.28**%**	1.06	[0.80, 1.42]	0.734
	Girls	79/55.70%	113/61.95**%**	1.11	[0.80, 1.55]	0.734
9	Boys	77/57.14%	102/53.92**%**	0.94	[0.72, 1.23]	0.734
	Girls	57/59.65%	85/72.94**%**	1.22	[1.02, 1.46]	0.057
10	Boys	128/56.25%	82/69.51**%**	1.24	[1.06, 1.43]	**0.020**
	Girls	71/63.38%	59/79.66**%**	1.26	[1.01, 1.56]	0.062
11	Boys	95/62.11%	143/75.52**%**	1.22	[1.02, 1.45]	0.057
	Girls	77/66.23%	75/93.33**%**	1.41	[1.10, 1.81]	**0.020**

Suspected myopia was defined as unaided distance visual acuity < 5.0 (Chinese standard logarithmic visual acuity chart) in either eye. PRs and 95% CIs were obtained from modified Poisson regression contrasts, with class-level cluster-robust standard errors. *q*-values were adjusted using the Benjamini–Hochberg false discovery rate within this outcome.

## 4 Discussion

The study findings underscore the significant changes in children's physical fitness levels during the COVID-19 pandemic, with notable sex- and age-specific differences observed across various physical fitness tests. They also reveal a marked increase in the prevalence of myopia among certain age groups during the pandemic, fueling further concerns about the impact of pandemic-related lifestyle changes on children's visual health. These results reflect the broader global trend of reduced physical activity during lockdowns and restrictions, but with some intriguing variations that warrant further investigation.

In the 50 m sprint, the primary year effect was not statistically significant, and no within-stratum 2020 vs. 2019 contrast remained significant after BH-FDR correction. Therefore, the present findings do not provide strong statistical evidence of pandemic-related changes in sprint performance. Generally, sprint speed in children aged 6–11 improves with age, largely due to increases in stride length and muscle development (30). Although pandemic-related restrictions may have influenced children's physical activity patterns, the current results suggest that sprint performance remained relatively stable across the two time points. Previous studies conducted during the COVID-19 pandemic have reported inconsistent findings regarding changes in running performance. For example, a study in Japan (31) observed slower 25 m run times among preschool children during the pandemic period. These discrepancies may reflect differences in age groups, environmental contexts, or the degree of restriction experienced. Running remains a fundamental motor skill encompassing locomotion, balance, and coordination. In preparation for potential future disruptions to structured physical education, maintaining opportunities for regular practice of fundamental movement skills, including running and jumping, remains important for supporting healthy motor development.

This study revealed distinct age- and sex-specific variations in flexibility during the pandemic. After BH-FDR correction, significant declines in sit-and-reach performance were observed in boys aged 6 years and girls aged 6–7 years, whereas girls aged 10 years showed higher flexibility in 2020. These findings indicate heterogeneous age- and sex-specific changes rather than a uniform decline across all boys or all older girls. Notably, the FDR-significant declines were concentrated in younger strata, suggesting that early primary-school ages may have been particularly sensitive to pandemic-related disruptions (Figure 2). Previous studies have consistently reported that girls demonstrate greater flexibility than boys during childhood, with such differences emerging as early as 6–8 years and widening with age (31). Superior flexibility has been observed in girls aged 6–8 years compared with boys of the same age (31), and similar patterns were noted among children aged 7–14 years (32). In contrast, flexibility differences by sex were found to be subtle in the youngest age group (5–6 years) (33). National surveillance data from 2010 to 2019 indicated that flexibility, as assessed by the sit-and-reach test, showed an overall improvement among Chinese children and adolescents, with boys and girls exhibiting increases of 1.75% and 6.10%, respectively, between 2014 and 2019 (34). This suggests that prior to the pandemic, flexibility performance had remained stable or slightly improved, particularly among girls. In contrast, during the COVID-19 period, this study observed lower flexibility performance among boys aged 6–11 years and a mixed pattern among girls compared with the 2019 survey wave, suggesting that flexibility patterns in 2020 differed from the previously reported improving trends. These between-year differences may be associated with changes in physical activity opportunities and stretching-related activities during periods of home confinement reported in previous literature (35, 36). However, these behavioral factors were not directly assessed in the present study. The findings suggest heterogeneous associations between the 2020 survey wave and flexibility outcomes according to sex and age, highlighting the importance of monitoring flexibility development, particularly among younger children and boys.

Our findings are consistent with previous studies reporting between-year differences in physical fitness outcomes among primary school children during the COVID-19 period, as demonstrated in previous studies from Austria (ages 7–10), Japan (ages 6–7), and Portugal (age 7) (19, 20, 37). However, few studies have objectively measured the physical fitness of primary school students across a wide age range. Notably, our study is the first to analyze flexibility across age groups among primary school children.

In the present study, rope-jumping performance improved significantly in most age groups during the pandemic period, whereas boys aged 6 and 10 years showed non-significant declines. These findings suggest that rope-jumping ability may have been maintained or even enhanced in many children despite broader restrictions on structured physical activity.

Rope jumping is a complex motor task requiring coordination, rhythm control, neuromuscular timing, and lower-limb muscular endurance. Although sprint speed and muscular power are associated with rope-jumping ability, performance in rope jumping does not depend exclusively on short-distance running speed. Therefore, the absence of significant changes in 50 m sprint performance does not contradict the observed improvements in rope-jumping performance. Previous studies have reported positive associations between speed, strength, and endurance with rope-jumping performance in late childhood (10–12 years) (38), further supporting the multifactorial nature of this motor skill.

Because rope jumping requires minimal space and equipment and was widely recommended in home-based physical education programs during school closures (4, 39), greater participation in home-based rope-jumping activities reported during the COVID-19 period may be associated with the relatively higher rope-jumping performance observed in certain age groups (7–9 and 11 years). However, participation in rope-jumping activities was not directly assessed in the present study. In contrast, younger children, particularly 6-year-olds, are still developing fundamental motor coordination and may rely more heavily on structured school-based physical education environments (40). Reduced opportunities for guided motor skill practice during school closures may also be associated with the absence of improvement observed in this age group. Sex differences may also reflect variations in activity preferences and engagement patterns, as boys are more likely to prefer competitive and group-based activities that were less accessible during lockdown periods (41). However, these interpretations remain speculative, as behavioral data on actual activity participation were not available in the present study. Overall, the findings suggest that pandemic-related changes in children's physical fitness were task-specific and heterogeneous across developmental stages and sex, rather than reflecting uniform declines in overall physical capacity.

The prevalence of suspected myopia (defined based on unaided distance visual acuity screening) showed an overall increase from 2019 to 2020; however, the between-year contrast was heterogeneous across age and sex. In the primary model, the year effect was significant (*p* < 0.001) with evidence of age-dependent differences (year × age *p* < 0.01). After controlling for multiple comparisons within this outcome using the Benjamini–Hochberg false discovery rate, PRs comparing 2020 with 2019 were significantly higher among girls aged 7 years and 11 years, and among boys aged 10 years. In contrast, boys aged 6 years showed a lower prevalence in 2020 than in 2019 (Table 2). The sensitivity analyses also suggest that the observed increase in suspected myopia was unlikely to be explained by the sex composition difference at age 11. In an age 11 specific standardization check, standardizing the 2020 sex distribution to the 2019 sex distribution slightly increased, rather than decreased, the estimated age 11 prevalence ratio. Thus, the higher proportion of boys among children aged 11 years in 2020 did not explain the observed increase in suspected myopia.

This pattern is consistent with previous literature suggesting that lifestyle changes during the COVID-19 period, including reduced outdoor exposure and increased near work and screen-based activities, may be associated with reduced visual health in children. Although we did not directly measure outdoor time or screen time, it is plausible that reduced opportunities for outdoor play and increased near work during home confinement contributed to the observed increases in several subgroups. Previous experimental and epidemiological studies have proposed that outdoor light exposure may protect against myopia through dopamine-mediated pathways that inhibit excessive axial elongation, whereas prolonged near work may increase accommodative demand and be associated with myopic progression (42).

Although outdoor time, near work, and screen exposure were not directly measured in the present study, previous studies have suggested that these factors may be associated with increased prevalence of suspected myopia during the COVID-19 period. Additionally, increased screen time during this period may have contributed to the increased prevalence of myopia (43). Public health initiatives focusing on eye health, including regular eye examinations and encouraging outdoor activities, are crucial in mitigating this issue. Moreover, parents and teachers can limit screen time and incorporate physical activities (including those related to physical training) into children's daily routines while complying with infection prevention rules (44, 45).

Based on the observed associations in this study together with previous public health evidence, preventive health strategies for children may benefit from emphasizing lifestyle management, we recommend that public health policy interventions, particularly in preventive health measures for children, prioritize lifestyle management as a key intervention area. In terms of weight management and mitigating the likelihood of obesity (46), it is recommended that children engage in at least 60 min of moderate to vigorous physical activity daily, limit high-sugar snack intake, and encourage family meals (47, 48). To minimize the risk of myopia progression and promote vision health, it is recommended that children engage in at least 2 h of outdoor activities daily, limit continuous near-screen time to ≤ 30 min, and incorporate visual rest breaks during learning or reading (49, 50). A key finding of this study is that children's jump rope performance significantly improved during home isolation, suggesting that indoor exercise is highly feasible. Based on this, the following strategies can be promoted in practice: (1) include home exercise forms such as jump rope, calisthenics, indoor aerobic exercise, in school physical education courses; and (2) develop and promote indoor exercise guidance materials for families.

This study had several limitations. First, the study employed a single-school convenience-sample design in a rural primary school in Lishui City, Zhejiang Province, China. Therefore, the findings should be interpreted as context-specific evidence and may not be generalizable to urban populations or the broader national population. Larger multi-center studies are needed to confirm the generalizability of these findings. Second, because the study was conducted within a single school across two consecutive years, some participants assessed in 2020 may also have participated in the 2019 assessment. Therefore, the age-stratified cohorts were not fully independent, which may have influenced the regression estimates and standard errors. Age was classified in yearly categories rather than by month, which may have reduced precision in age-stratified comparisons. Third, suspected myopia was determined using unaided distance visual acuity screening in accordance with national procedures; however, cycloplegic refraction was not performed, which may have led to misclassification and potential overestimation of myopia prevalence (43). Therefore, some participants may have been erroneously classified as myopic. Fourth, although the Discussion refers to potential mechanisms such as outdoor light exposure, screen time, near-work exposure, accommodative demand, and dopamine-related pathways, these variables and mechanisms were not directly assessed in the present study and were interpreted based on prior literature. Fifth, we did not investigate the potential effects of the frequency or type of physical activity at school or home. In addition, potential cohort confounding, examiner-related variation, equipment-related variation, and other secular changes between 2019 and 2020 could not be completely ruled out. Finally, the cross-sectional design of this study precludes causal inferences.

## 5 Conclusions

This study identified heterogeneous changes in children's physical fitness, suspected myopia, and BMI during the COVID-19 pandemic in China, with clear age- and sex-specific patterns. BMI increased significantly among children aged 6–9 years, and suspected myopia rose in several age–sex strata, particularly among girls aged 7 and 11 years and boys aged 10 years. In contrast, physical fitness outcomes were task-specific. While flexibility declined in selected age groups and sprint performance remained largely stable, rope-jumping performance improved significantly in most age groups, with only non-significant declines observed among boys aged 6 and 10 years. Overall, these findings suggest that pandemic-related lifestyle changes did not uniformly impair children's physical capacity but instead produced domain-specific and developmentally patterned effects. The observed heterogeneity underscores the importance of age- and sex-sensitive monitoring and intervention strategies to address the long-term impacts of large-scale public health disruptions on child health.

## Ethic statement

The study protocols were approved by the Waseda University Ethical Committee (No. 2023-HN051). Written informed consent was obtained from the guardians of all participants prior to data collection.

## Data Availability

The raw data supporting the conclusions of this article are not publicly available due to privacy and ethical restrictions. Further inquiries can be directed to the corresponding author and will be considered upon reasonable request.
